# Meiotically Stable Natural Epialleles of *Sadhu,* a Novel Arabidopsis Retroposon

**DOI:** 10.1371/journal.pgen.0020036

**Published:** 2006-03-17

**Authors:** Sanjida H Rangwala, Rangasamy Elumalai, Cheryl Vanier, Hakan Ozkan, David W Galbraith, Eric J Richards

**Affiliations:** 1 Department of Biology, Washington University in St. Louis, St. Louis, Missouri, United States of America; 2 Department of Plant Sciences, University of Arizona, Tucson, Arizona, United States of America; 3 Department of Biological Sciences, University of Nevada, Las Vegas, Nevada, United States of America; University of Wisconsin, United States of America

## Abstract

Epigenetic variation is a potential source of genomic and phenotypic variation among different individuals in a population, and among different varieties within a species. We used a two-tiered approach to identify naturally occurring epigenetic alleles in the flowering plant Arabidopsis: a primary screen for transcript level polymorphisms among three strains (Col, Cvi, Ler), followed by a secondary screen for epigenetic alleles. Here, we describe the identification of stable, meiotically transmissible epigenetic alleles that correspond to one member of a previously uncharacterized non-LTR retroposon family, which we have designated *Sadhu*. The pericentromeric *At2g10410* element is highly expressed in strain Col, but silenced in Ler and 18 other strains surveyed. Transcription of this locus is inversely correlated with cytosine methylation and both the expression and DNA methylation states map in a Mendelian manner to stable *cis*-acting variation. The silent Ler allele can be converted by the epigenetic modifier mutation *ddm1* to a meiotically stable expressing allele with an identical primary nucleotide sequence, demonstrating that the variation responsible for transcript level polymorphism among Arabidopsis strains is epigenetic. We extended our characterization of the *Sadhu* family members and show that different elements are subject to both genetic and epigenetic variation in natural populations. These findings support the view that an important component of natural variation in retroelements is epigenetic.

## Synopsis

Differences among biological strains or individuals in a population can arise either from changes in DNA sequence (genetic) or in the packaging of DNA within the nucleus independent of DNA sequence (epigenetic). Both types of changes can alter gene activity, although epigenetic variation is often thought to be transient and unable to affect inherited differences among organisms. The authors compared the amount of RNA transcripts—a measure of gene activity—from a comprehensive set of genes among different strains of the flowering plant Arabidopsis. This approach led to the discovery of a novel family of DNA sequences, termed *Sadhu,* which show both genetic and epigenetic variation in gene activity. Alternative epigenetic states of one *Sadhu* element were created using mutants defective in epigenetic regulation. Both natural and induced epigenetic states were inherited. These results demonstrate that inherited differences among natural populations can be caused by epigenetic as well as genetic differences. *Sadhu* elements are a type of transposon, a class of DNA sequences that can move from one position in the genome to another. Epigenetic variation in gene activity of transposons modulates their movements within the genome and can influence genome diversification and evolution.

## Introduction

Epigenetic information in the form of differential DNA methylation, histone modification, and chromatin packaging is important for the management of large, complex eukaryotic genomes [1,2]. The stability of both animal and plant genomes depends heavily on epigenetic modification of repetitive DNA, including transposable elements and long tandem arrays of short repeats. For example, loss of genomic DNA methylation leads to meiotic defects [3], chromosome decondensation [4–7], transcription of previously quiescent transposons [8–11], and increased mutagenesis via DNA rearrangements [12]. Another component of genome stability is the integrity of epigenetic states that cement transcription rates of individual genes. These epigenetic states can be remarkably stable and transmitted faithfully through mitosis [13,14]. Alterations in these states form epigenetic alleles, or “epialleles,” that lead to aberrant gene expression. The accumulation of epialleles in somatic tissues is now recognized as an important component of human carcinogenesis (e.g., tumor suppressor gene silencing) [15–17] and degenerative diseases associated with aging, such as atherosclerosis [18,19]. Transmission of epialleles is not restricted to mitotic divisions, but can also occur between generations of organisms [20–24], thereby mimicking traditional genetic mutations. This situation may be commonplace in plants where DNA methylation can be inherited through meiosis with high fidelity [25–28]. These findings raise the possibility that a significant portion of inherited information may be epigenetic and partially independent of the genetic sequence.

We are interested in determining the contribution of meiotically stable epigenetic alleles in the generation of genomic and phenotypic diversity. We exploited the availability of different accessions of the flowering plant Arabidopsis thaliana to evaluate the significance of the epigenetic component of inheritance in natural populations. We previously reported variation among Arabidopsis accessions in 5-methylcytosine (5mC) levels in the long tandem arrays of the major ribosomal RNA gene repeats [27]. Further, we showed that cytosine methylation patterns in inter-strain crosses are controlled by a combination of epigenetic inheritance of parental methylation patterns and the action of *trans*-acting loci [27,29]. Here, we describe a screen for natural epigenetic variation in cytosine methylation that is associated with transcript level polymorphisms among strains of Arabidopsis. This screen has led to the discovery of a new class of non-autonomous retroposons that are subject to epigenetic variation among natural accessions.

## Results

### Screen for Candidate Natural Epigenetic Variants

We set out to discover naturally occurring epialleles by identifying transcripts that were, first, differentially expressed in Arabidopsis accessions derived from wild populations and, second, whose transcription activity correlated with epigenetic state. An Arabidopsis long-oligo array containing approximately 26,000 predicted gene targets was hybridized with cDNA synthesized from whole-seedling RNA from the accessions Col, Ler, and Cvi. 279 loci were found to be differentially expressed (ANOVA *p*-values <0.1) with changes greater than 2-fold in pair-wise comparisons of these accessions. Here, we describe our characterization of one locus, *At2g10410,* identified in this screen for natural epialleles.

### *At2g10410* Is Subject to Natural Variation That Maps in *cis*

**Figure 1:**
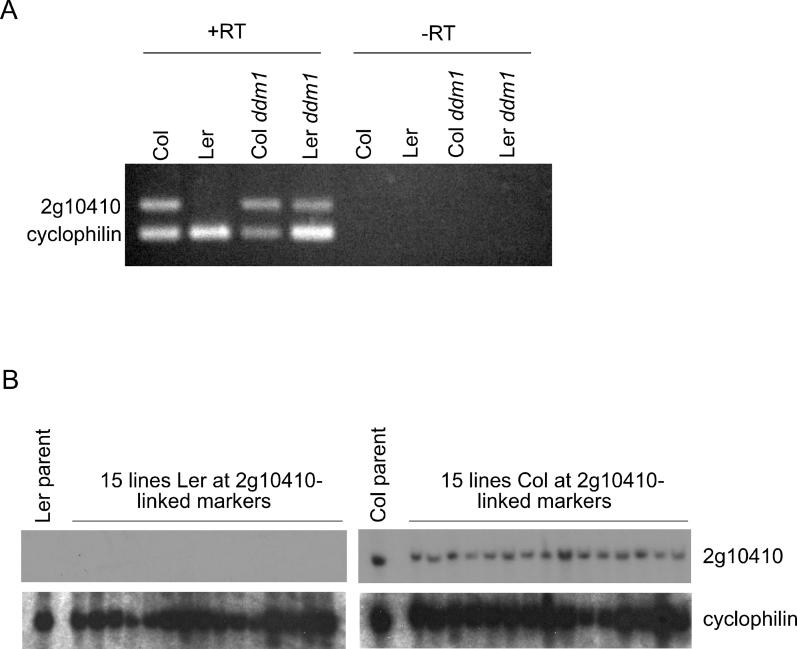
*At2g10410* Is Differentially Expressed in the Arabidopsis Accessions Col and Ler (A) RT-PCR of *At2g10410* expression in Col, Ler, Col *ddm1–1,* and Ler *ddm1–2*. Cyclophilin is shown as an internal control for amplification. (B) RNA gel blots of Col/Ler recombinant inbred lines. Lines homozygous for Col alleles at *At2g10410*-linked markers express the locus, while lines homozygous for Ler alleles at linked markers do not express *At2g10410*. All lanes were included on the same filter. The filter was rehybridized with a cyclophilin probe as a loading control.

The microarray data indicated lower expression of *At2g10410* in Ler and Cvi compared with Col. Robust transcription of *At2g10410* in Col was confirmed by RT-PCR (Figure 1A, Table 1) and RNA gel blot analysis (Fig 1B, unpublished data); we did not detect expression of this locus in the Cvi and Ler accessions. Expression of the Col *At2g10410* allele was corroborated by the massively parallel signature sequencing (MPSS) cDNA project (http://mpss.udel.edu/at) [30], which indicated a transcript level on the order of 180 transcripts per million (tpm). In addition, whole-genome transcriptome analysis in Col using a high-density oligonucleotide array [31] revealed that this locus is the most highly expressed feature within a 600-kb window of the transposon-rich pericentromeric region of Chromosome 2. The lack of detectable *At2g10410* expression in Cvi and Ler is not caused by the absence of this locus, which could be amplified from Ler and Cvi genomic DNA templates (Table 1). We also examined 22 additional Arabidopsis accessions and detected the presence of the *At2g10410* locus in the majority of these natural strains (Table 1). However, of the accessions containing the locus, we detected expression by RT-PCR in only three (Col, N13, and Pu2–7). These data demonstrate the existence of natural variation in *At2g10410* expression.

**Table 1:**
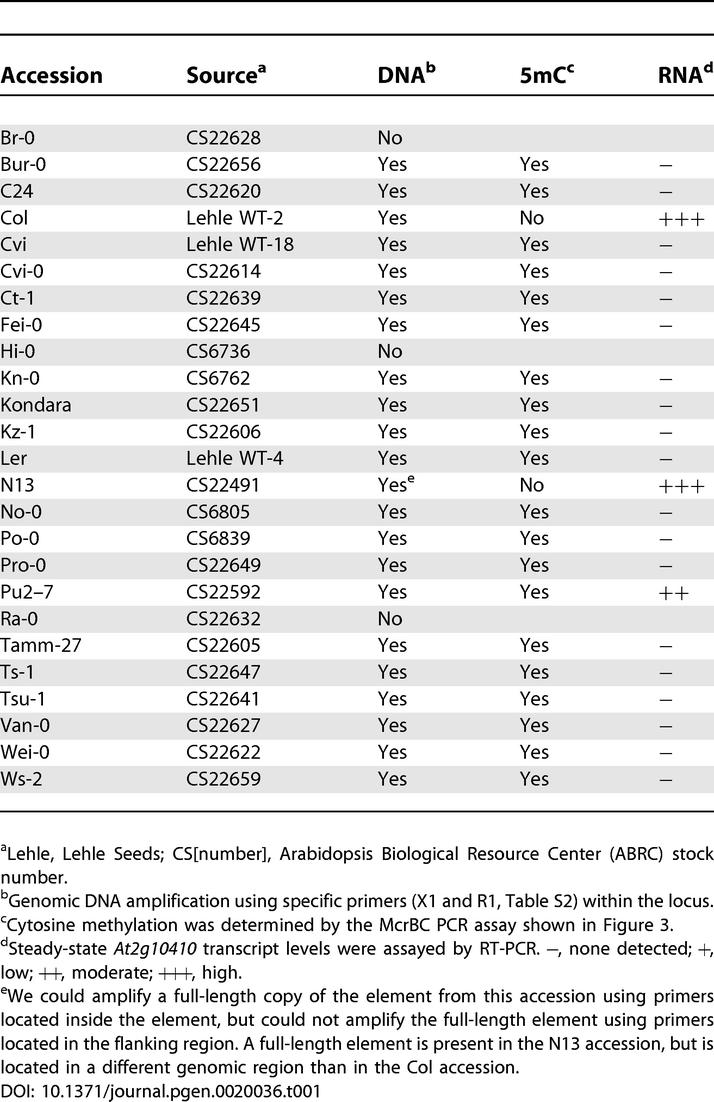
Natural Variation of *At2g10410* Structure, Cytosine Methylation, and Expression

We examined a Col/Ler recombinant inbred (RI) population [32] to determine whether *At2g10410* expression states mapped in *cis* or to a *trans*-acting transcriptional modifier. We selected fifteen RI lines that were Col homozygous and fifteen lines that were Ler homozygous at markers flanking the *At2g10410* locus. RNA gel blot analysis indicated that all the lines containing the Col allele expressed *At2g10410,* while all the lines containing the Ler allele were silenced at this locus (Figure 1B). These results argue against an unlinked Col or Ler specific factor that influences expression at *At2g10410*. Instead, expression of this locus maps in *cis* and is stable through the eight generations of self-fertilization used to generate the recombinant inbred lines.

### *At2g10410* Is a Unique, Non-Coding Sequence That Arose by Retroposition

**Figure 2:**
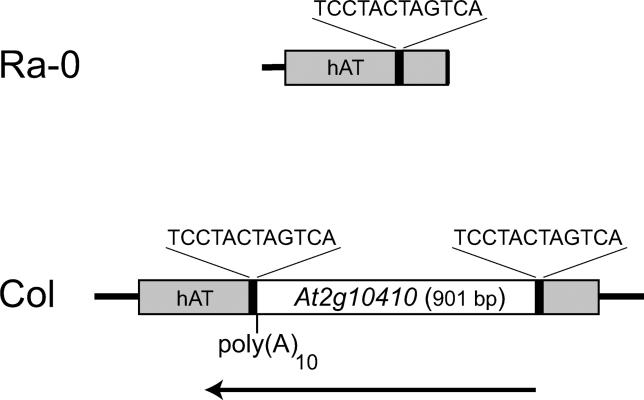
Structure of *At2g10410* in Two Accessions *At2g10410* in Col is inserted within a sequence that is 93% identical to a hAT10 (1536 to 2399 coordinates) DNA transposase gene (grey box, hAT). The poly(A) tract and target site duplication in Col are shown. The *At2g10410* transcript is indicated by the arrow. The Ra-0 accession lacks the *At2g10410* insertion, poly(A) tract, and target site duplication.

*At2g10410* does not contain a long open reading frame and is not significantly similar to any known protein-coding sequence in any of the plant, animal, bacterial, or viral sequence collections. There is likewise no obvious sequence or structural similarity to any known non-coding RNA gene. *At2g10410* is composed of 901 bp of unique sequence inserted within a hAT family DNA transposase pseudogene (Figure 2). EST data suggest a polyadenylated full-length transcript of 1,054 bp, ending 130 bp within the flanking transposase sequence. There is also a poly(A) stretch of ten nucleotides at the boundary of the hAT transposon and unique sequence of this element. In addition, the entire unique sequence is flanked by a direct duplication of 12 nucleotides of hAT sequence. The corresponding region in the Ra-0 accession (Table 1) contains a continuous hAT transposase pseudogene, lacking the *At2g10410* unique sequence, the poly(A) tract, and the 12 nucleotide duplication (Figure 2). These genomic structure comparisons support a model in which the *At2g10410* gene in the Col accession derived from a polyadenylated RNA precursor that has retrotransposed into the ancestral genomic sequence, represented by the Ra-0 allele.

**Figure 3:**
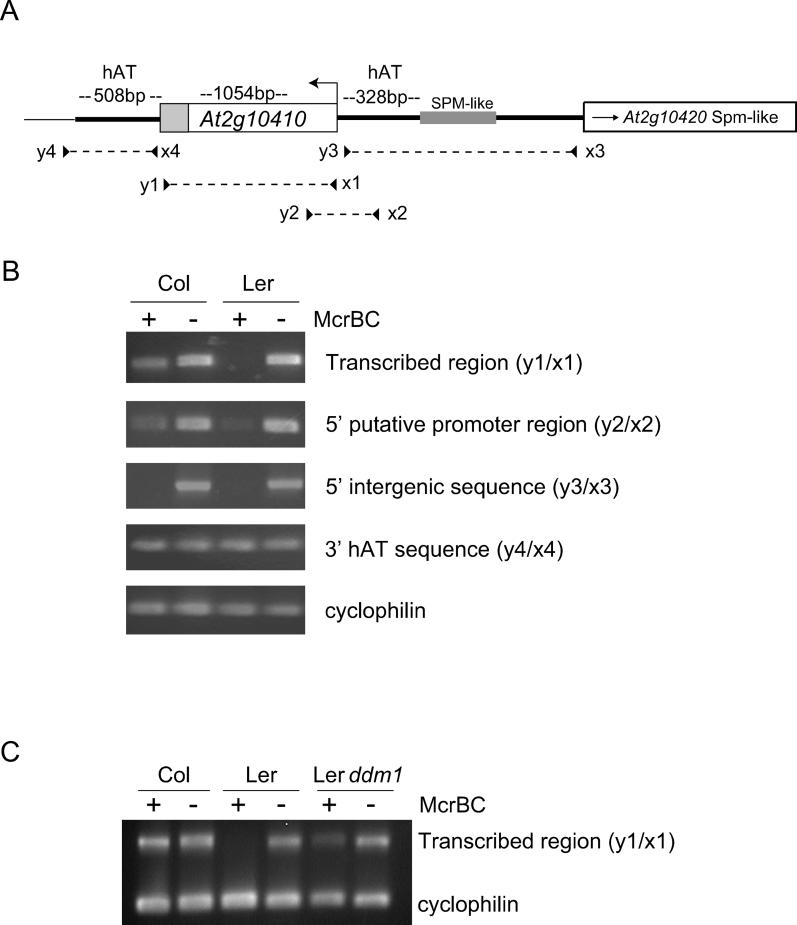
Analysis of Cytosine Methylation at *At2g10410* by McrBC Digestion followed by PCR (A) Diagram of the *At2g10410* locus in Col including nearby repetitive elements and intergenic regions. Arrow indicates start and direction of transcription. The positions of primers used for PCR analysis are indicated as triangles. (B) PCR amplification of genomic DNA +/− McrBC digestion. Cyclophilin is shown as an amplification control. (C) McrBC PCR of the *At2g10410-*transcribed region in Col, Ler, and Ler *ddm1–2* genomic DNA samples. Cyclophilin is shown as an internal amplification control.

### Genetic and Epigenetic Variation of *At2g10410*

We explored the possibility that genetic differences between the expressed Col *At2g10410* allele and the unexpressed Ler allele are responsible for transcription level differences. We determined that the nucleotide sequence of approximately 1.7 kb of Ler genomic DNA encompassing the *At2g10410* locus was 98.6% identical to the Col reference genome sequence (Figure S1). The polymorphism frequency observed in the transcribed region was similar to that in the 5′ and 3′ non-transcribed flanking regions. No large indels or rearrangements were observed in our comparison of this region between the Col and Ler accessions and only two SNPs exist within +/− 150 bp of the transcription start site (Figure S1).

We next investigated cytosine methylation at *At2g10410* using a PCR-based assay that monitors digestion of genomic templates by the methylation-requiring restriction enzyme McrBC [33]. In every natural accession we examined, transcriptionally silent alleles at this locus were also methylated (Table 1). By contrast, both the expressed N13 and Col alleles of *At2g10410* are hypomethylated within the transcribed region. We observed one exception to the trend that methylated sequences are silent: the Pu2–7 *At2g10410* allele was both methylated and expressed (Table 1). We also examined cytosine methylation at *At2g10410* in the Col/Ler RI lines described above. The *At2g10410* locus was hypomethylated in all RI lines containing an expressed Col allele, while the locus was methylated in all lines carrying the silent Ler allele (unpublished data). Therefore, cytosine methylation was strictly correlated to the expression state of the Col and Ler allele. Moreover, these data suggest that the parental cytosine methylation state of the two alleles is stably inherited through the multiple generations required to construct the independent RI lines.

We also examined cytosine methylation flanking the *At2g10410* transcribed region in Ler and Col accessions to determine the boundaries of the differential methylation states of these alleles. Alleles from both of these accessions had comparable methylation levels in the regions 1 kb upstream or 400 bp downstream of the transcript, even though they were differentially methylated within the gene (Figure 3A and 3B). These data indicate that the differential methylation that we observed between silenced and expressed accessions is limited to the region of transcription.

A higher resolution map of cytosine methylation of the *At2g10410* locus was constructed using bisulfite-mediated genomic sequencing [34] of a 380-bp region encompassing the start of transcription. In the Col allele a boundary of cytosine methylation coinciding with the transcription start site was observed; the region downstream of transcription was almost entirely free of methylation. On the other hand, the Ler allele was methylated both downstream and upstream of transcription (Table 2; Figure S2). In the Ler allele, the methylation occupancy at CpG sites was high (~90%), and less methylation was observed at cytosines at CpHpG (~30%) or asymmetrical CpHpH sites (~14%). These data corroborate the McrBC-PCR results and confirm that cytosine methylation is absent from the transcribed region in the expressed Col *At2g10410* allele.

**Table 2:**
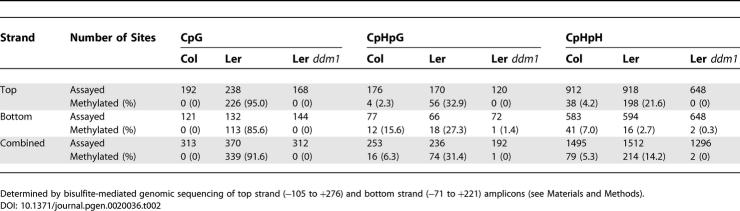
Number of Cytosines Methylated at the *At2g10410* Locus in Col, Ler, and Ler *ddm1–2* Plants

### DNA Hypomethylation of the Ler *At2g10410* Locus Induces Ectopically Expressing and Meiotically Stable Epialleles

Having established that cytosine methylation correlates with the expression state of *At2g10410* in different strains, we asked whether the silent Ler allele could be reactivated by manipulating DNA methylation of the locus. We first treated Ler seedlings with the cytosine-DNA-methyltransferase inhibitor 5-aza-deoxycytidine [35,36] and observed ectopic transcription of the *At2g10410* locus (unpublished data). Next, we monitored expression of this locus in Ler strains carrying DNA hypomethylation mutations: *met1–1* (disrupting the major Dnmt1-class CpG “maintenance” methyltransferase [37,38]) or *ddm1–2* (disrupting a SNF2-class ATP-dependent nucleosome remodeling protein gene [28,39]). As shown in Figure 1A, loss of *DDM1* function leads to ectopic expression of the Ler *At2g10410* allele; similar results were observed for the *met1–1* mutant (unpublished data). McrBC-PCR suggested that the expressing *At2g10410* allele in the Ler *ddm1–2* background is hypomethylated relative to the silenced allele in Ler wild-type (Figure 3C). Bisulfite-mediated genomic sequencing of the *At2g10410* locus in Ler *ddm1–2* individuals confirmed complete loss of cytosine methylation in all sequence contexts (Table 2). RNA gel blots indicated that the ectopic *At2g10410* transcript in Ler *ddm1–2* plants is approximately the same size as the transcript in Col (unpublished data). We determined the DNA sequence from 245 bp upstream to 650 bp downstream of transcription in the Ler *ddm1–2* mutant and found no differences from the Ler wild-type sequence (unpublished data). These findings indicate that the *ddm1–2* mutation did not alter the genetic information at the Ler *At2g10410* locus, but did change the DNA methylation and transcription states of the locus.

**Figure 4:**
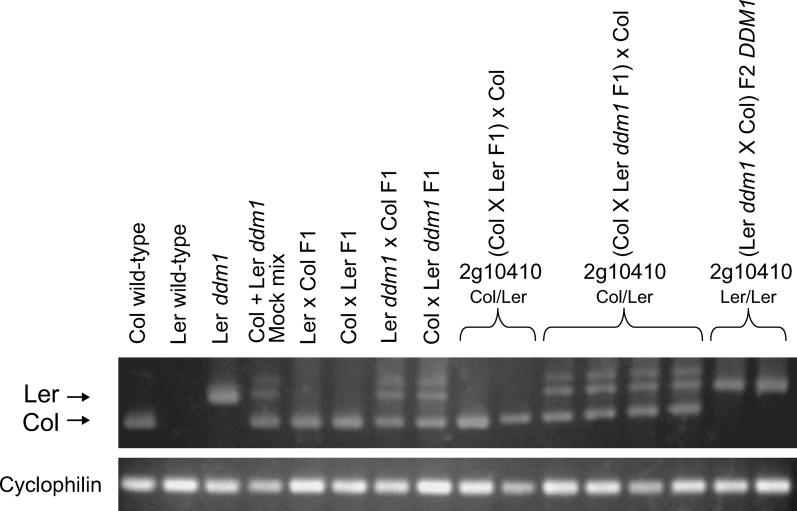
RT-PCR/CAPS (Cleaved Amplified Polymorphic Sequence) Detection of Allele-Specific Expression at *At2g10410* The Col allele is cleaved with *Bst*B1, generating a 480-bp fragment plus an undetected 70-bp fragment, while the Ler allele is uncleaved (550 bp). *At2g10410* is not expressed in Ler wild-type, but is expressed in wild-type Col and Ler *ddm1* mutant, as shown in Figure 1. A mixture of cDNA templates from Col and Ler *ddm1* samples was used to illustrate the detection of bi-allelic expression; note an additional higher molecular weight heteroduplex band (see Materials and Methods) in samples showing bi-allelic expression. A total of five Col × Ler heterozygous *At2g10410* BC1 and ten Col × Ler *ddm1–2* heterozygous BC1 individuals were examined; all looked identical to the representative individuals shown. In addition, we examined two *DDM1* ^+/+^ F2 individuals homozygous for the Ler allele at *At2g10410* that resulted from self-pollination of a Ler *ddm1* × Col F1 individual. RT-PCR amplification of cyclophilin transcripts is shown as a control.

To see whether *ddm1*-induced expression of the Ler *At2g10410* allele was stable in the presence of a functional *DDM1* allele, we outcrossed a Ler *ddm1–2* individual to wild-type Col. F1 hybrids generated from reciprocal crosses maintain expression of both the Col and Ler alleles (Figure 4). By contrast, there was no expression of the Ler allele in F1 hybrids of a control cross between wild-type Col and Ler individuals. Therefore, parental expression states at *At2g10410* are faithfully maintained in an inter-strain cross, and there is no evidence for a Col or Ler specific *trans*-acting modifier. When F1 *At2g10410* ^Col/Ler^ hybrids were backcrossed to the Col parent strain (→ BC1), individuals heterozygous for *At2g10410* alleles from both Col and Ler parents continued to maintain the expression states inherited from the original parents. Ten Col × [Col × Ler *ddm1–2*] BC1 individuals examined showed bi-allelic expression, while five Col ×[Col × Ler wild-type] BC1 individuals examined showed expression of the Col allele only (Figure 4). We note that Col × [Col × Ler *ddm1–2*] BC1 individuals must be either heterozygous or homozygous wild-type at the *DDM1* locus. We conclude that ectopic expression of the Ler allele can be maintained in the absence of a homozygous *ddm1–2* mutation. Moreover, we examined two F2 individuals from the Col × Ler *ddm1–2* cross that were homozygous wild-type at the *DDM1* locus and homozygous Ler at the *At2g10410* locus. Both of these F2 individuals, which no longer carried the *ddm1–2* mutation or the Col *At2g10410* allele, persisted in their expression of the Ler *At2g10410* allele (Figure 4). These data indicate that expression states at *At2g10410* can be modified in a *ddm1* mutant background and that once established, ectopic expression states can be inherited as meiotically stable epialleles. Taken together with the RI mapping results detailed above, we conclude that silenced or active expression states at *At2g10410* behave as stable epialleles.

### *At2g10410* Is a Member of a Previously Uncharacterized Non-Autonomous Retroposon Family

**Figure 5:**
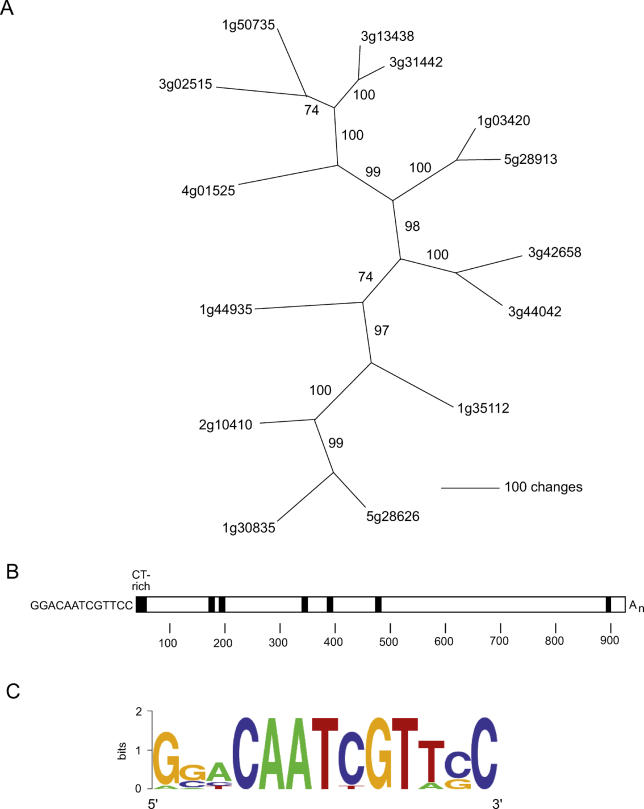
*Sadhu* Retroposon Family Members in the Arabidopsis Col Genome (A) An unrooted phylogram of 14 full-length *Sadhu* elements built using a maximum parsimony algorithm. Bootstrap values are shown (100 bootstrap replicates). (B) Diagram of consensus sequence structure of a full-length *Sadhu* element based on ClustalX gapped alignment. Black boxes indicate short blocks (10–20 nucleotides) of high sequence conservation, including a CT-rich block toward the 5′ end. The majority of elements end with a poly(A) tract (Table 3). (C) A conserved 13-bp sequence at the predicted 5′ boundary of the element is illustrated as a logo diagram based on alignment of the 14 homologs shown in (A) [72].

After establishing that *At2g10410* was a novel retroposon subject to natural epigenetic variation, we searched the available Arabidopsis genomic sequence from strain Col for related sequences. Fourteen other sequences in the Col genome share 55%–75% identity over the ~850–900 bp of *At2g10410* unique sequence (Table 3; Figure 5A). Consistent with being generated through retroposition, 13 of the 14 homologs contain 3′ poly(A) tracts, while eight feature recognizable target site duplications of at least ten nucleotides (Table 3). The 3′ target site duplication always occurs adjacent to the poly(A) tract; however, the other target site duplication occurs anywhere between 8 bp and 75 bp 5′ of the conserved sequence. This observation suggests that the 5' boundary of retroposition can vary in both length and sequence. Apart from conservation of genomic structure, there are several short stretches of high DNA sequence identity among the 14 homologs separated by areas of little or no similarity (Figure 5B). Of note, there is a 13 nucleotide sequence (consensus 5′-GGACAATCGTTCC-3′) near the start of *At2g10410* transcription that is followed by a 10–20 nt CT-rich region (Figure 5B and 5C). DNA sequence conservation is restricted to the unique transcribed sequence of *At2g10410;* there is no similarity among family members in the immediate upstream or downstream flanking genomic regions. These features suggest that each member has retroposed independently into its flanking genomic region without obvious selection for a particular region. Because none of these homologs contains an ORF with similarity to a transposase-related protein, these sequences fit the criteria of a family of previously uncharacterized non-autonomous retroposons. We have named these sequences *Sadhu* elements, after the Sanskrit term for ascetic holy men who have renounced society.

**Table 3:**
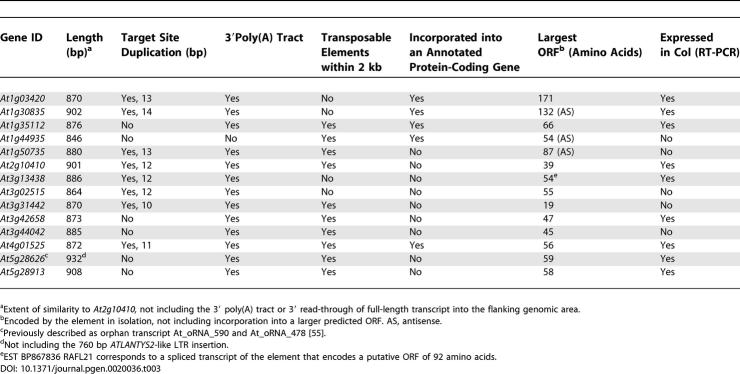
Features of 14 Full-Length Members of the *Sadhu* Element Family in the Col Genome

In addition to the 14 family members described in Table 3, there are 25 sequences of ~175–750 bp in the Arabidopsis Col genome that have similarity to the 5′, 3′, or an internal section of the full-length *Sadhu* elements (Table S1). We noted the presence of truncated 3′ elements, some of which contain poly(A) tracts and target site duplications. Such structures are predicted to arise from reverse transcription that did not proceed to the 5′ end of the transcript prior to transposition. Some of these truncated sequences are 99% identical to their closest full-length *Sadhu* element, with no flanking sequence similarity shared among these elements. These observations indicate that the closely related *Sadhu* elements did not arise by recent segmental duplication but represent independent recent retroposition events.

Ten of the 14 full-length *Sadhu* elements and 12 of the 25 truncated elements are located within the vicinity of other repetitive elements, such as transposons (Table 3, Table S1; see Figure S3 for chromosome distribution). Many of these are integrated within transposons; *At5g28626* is disrupted by an *ATLANTYS2*-like retrotransposon LTR sequence, while *At2g10410, At3g44042,* and *At3g31442* are embedded within DNA transposons. Despite this preference for integration near repetitive environments, transcription of nine of the 14 full-length *Sadhu* elements is detected in the Col accession by RT-PCR (Table 3). In addition, although the *Sadhu* elements are by themselves non-coding, five homologs have been annotated within putative protein coding genes (Table 3). EST data confirm that one of these, *At1g30835,* is in fact transcribed in antisense orientation to other *Sadhu* elements and encodes a 132 amino acid predicted protein. Two additional family members encode ORFs greater than 75 amino acids. The amino acid sequences encoded by these ORFs are independent of one another and do not resemble known protein coding sequences. In summary, most of the full-length *Sadhu* elements in Arabidopsis strain Col are expressed, and some members are candidates for generating potentially functional gene products.

### Several Other *Sadhu* Family Members Are Subject to Naturally Occurring Epigenetic Variation

We were interested in whether other members of the *Sadhu* retroposon family are, as with *At2g10410* characterized above, subject to natural variation in epigenetic transcriptional regulation. We focused on five full-length *Sadhu* elements—*At1g30835, At5g28626, At1g35112, At3g42658,* and *At3g44042,* which are closely related to *At2g10410* (Figure 5A). First, we screened a set of 25 accessions for the presence of that particular family member, as indicated by the ability to amplify these loci from genomic DNA templates (Table 4). Second, we evaluated gene expression of the loci verified to be present in the various accessions. Third, we monitored cytosine methylation status of the loci using the McrBC-PCR assay. There was considerable variation among the accessions for all three criteria at these five loci. For example, *At1g35112* was expressed at low levels in a few accessions, but transcriptionally silent or not amplified from genomic DNA in others. Some accessions contained methylated alleles at this locus, while others contained unmethylated alleles. The two accessions with the highest level of expression of this locus, Kz1 and N13, were both unmethylated. This result suggests that cytosine methylation may play a part in suppressing expression of some alleles of *At1g35112*. More notably, silencing was correlated with DNA methylation for most naturally occurring alleles of the loci *At5g28626, At3g42658,* and *At3g44042*. For these three *Sadhu* elements, the majority of the accessions containing hypomethylated alleles expressed these genes, while most accessions that contained methylated alleles were silent at these loci. It is likely that many of the alleles at these elements represent naturally occurring epialleles, as with the case for the Col and Ler alleles at *At2g10410*.

**Table 4:**
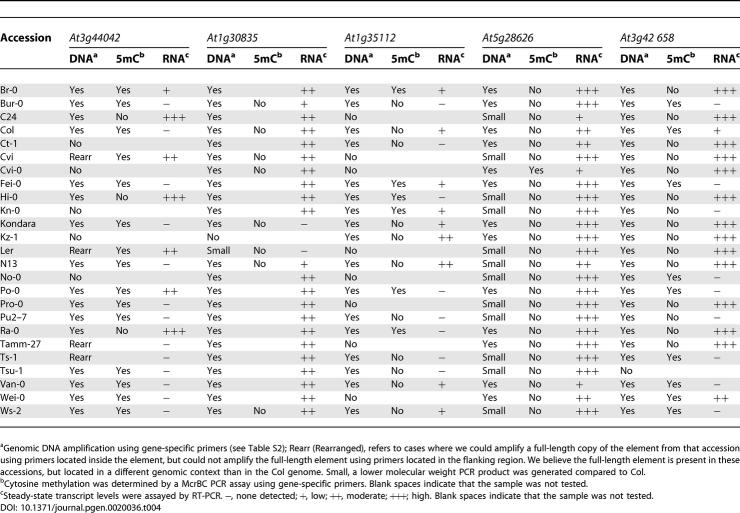
Natural Variation in Five *Sadhu* Elements among a Set of 25 Arabidopsis Accessions

## Discussion

We sought out transcript-level polymorphisms in natural populations that behaved as meiotically stable epialleles. Here we describe a locus, *At2g10410,* which is differentially expressed in different accessions of *Arabidopsis*. Silenced alleles are methylated predominantly at CpG sites over the transcribed region, while expressed alleles are correlated with an absence of cytosine methylation. Transcript level differences between the robustly expressed Col *At2g10410* allele and the silenced Ler allele map in *cis* in recombinant inbred lines. In addition, *ddm1*-induced ectopically expressed Ler alleles are meiotically stable upon introduction of the wild-type *DDM1* allele. Therefore, differentially expressing states of *At2g10410* in different Arabidopsis strains behave as stable epialleles.

*At2g10410* is a previously undescribed, unique, non-coding retroposed element. It is a member of a small family of such elements in Arabidopsis—*Sadhu* elements. These elements are typically ~900 bp long, with a poly(A) tract at the 3′ end and direct target site duplications. Because *Sadhu* elements do not share any sequence similarity to any known ORFs, they are unlikely to be processed pseudogenes, but are more reminiscent of SINE-class retroposons. However, *Sadhu* elements differ from canonical SINEs in that they are longer (>500 bp) and do not have recognizable RNA pol III promoter A or B boxes nor similarity to known SINE or SINE ancestral molecules such as tRNA, 5S rRNA or 7SL RNA (i.e., mammalian *Alu*) [40,41]. Therefore, *Sadhu* elements represent a family of novel retroelements.

*Sadhu* elements, like SINE retroelements, do not encode their own reverse transcriptase. SINE elements are thought to make use of LINE-encoded reverse transcriptase/endonuclease to create a DNA copy from RNA intermediates, which then inserts into the genomic DNA [40]. We do not find any reverse transcriptase- or transposase-encoding sequences related to the *Sadhu* elements in the available Arabidopsis Col genome sequence. We hypothesize that the *Sadhu* elements may be mobilized by LINE-encoded factors. It is unclear how exactly the LINE retrotransposition machinery recognizes its targets of transposition. SINE elements maintain significant conservation of motifs with their non-coding RNA ancestor molecules [40,42]. While *Sadhu* elements do not resemble SINEs or LINEs at the primary nucleotide level, they do show conservation of short motifs (Figure 5B) that may be functional in promoting mobilization. Although most *Sadhu* elements in the Col genome share only 60%–70% sequence identity, the presence of partial elements with 99% identity to one another (Table S1) suggests that mobilization is ongoing or has occurred recently in this family.

Mobilization of *Sadhu* elements by retroposition is expected to require expression of these elements into RNA intermediates. Indeed, most of the full-length *Sadhu* elements can be detected by RT-PCR in at least the Col strain (Table 3). Seven out of the 14 full-length *Sadhu* elements in Col are represented in the MPSS database at greater than 20 tpm in at least one tissue examined; *At2g10410* is expressed at greater than 100 tpm in most tissues. By contrast, we examined 170 annotated retroelements in the Col MPSS database [30], and found that less than ten were expressed at more than 20 tpm in any tissue examined (unpublished data). If the robustly expressed *At2g10410* is mobile or has been recently mobile, we would expect multiple copies of near identity in the genome. Preliminary analysis by Southern blot detects only one copy of this locus in the Col genome (unpublished data). This result suggests that transcription of *At2g10410* is not sufficient for transposition. Perhaps a reverse transcriptase source necessary to mobilize *Sadhu* elements is itself either nonfunctional or silenced in the Col strain. DNA transposons are mobile in *ddm1* mutants [9,10,43], and we are interested in exploring the possibility that LINE retrotransposition factors may become re-expressed in *ddm1* or other chromatin mutants, indirectly causing increased mobility of *Sadhu* elements.

The robust expression of full-length *Sadhu* elements, in contrast to the general non-activity of other retroelements in Arabidopsis, is puzzling for another reason—from what promoter are these elements transcribed? The 14 full-length *Sadhu* elements contain no similarity outside of the transcribed region, suggesting that the elements do not carry their own upstream conserved promoter or enhancer elements. Because the conserved motifs among the *Sadhu* elements downstream of transcription (Figure 5B and 5C) do not bear any resemblance to known RNA pol III promoters, it is possible that these sequences may instead represent novel, non-canonical RNA pol II or RNA pol III promoter elements. An alternative model is that transcriptionally active *Sadhu* elements have inserted near cryptic RNA pol II promoters. Pol II transcripts are polyadenylated, while pol III transcripts are typically not. In fact, there are oligo-d(T)-primed ESTs to *At2g10410* that do not originate from the poly(A) tract in the DNA sequence, supporting polyadenylation of this transcript. This evidence suggests that *At2g10410,* if not other *Sadhu* elements, may be transcribed from a flanking cryptic pol II promoter. We hypothesize that conserved downstream motifs in the *Sadhu* elements may act as enhancers to promote robust transcription of elements in a flexible variety of genomic contexts.

Our study of *At2g10410* and preliminary survey of other *Sadhu* family members suggest that there is considerable genetic, DNA cytosine methylation, and transcriptional variation in these elements among A. thaliana accessions (Table 1, Table 4). Across the *Sadhu* family members examined, there is a good correlation between cytosine methylation and lack of transcription. However, some exceptions exist. In the case of unmethylated alleles that are not expressed, genetic variation in promoter elements may be responsible for transcriptional inactivity. In instances where methylated alleles are transcribed, the expression level tends to be intermediate, consistent with partial silencing. Previous studies have highlighted the role of cytosine methylation in silencing of DNA transposons and retrotransposons [9–11,44–48]). However, this is the first report of strain-specific variation in both transcript abundance and cytosine methylation of a retroposon family.

The *Sadhu* family of retroelements represents a previously overlooked source of genetic and epigenetic variation in the genome. Barbara McClintock proposed over one half century ago that transposons (“controlling elements”) existing in different states or distinct genomic locations can differentially affect gene expression [49]. Recent studies have lent support to McClintock's view: epigenetic states at transposons can indeed affect the spread of transcription or silencing into neighboring coding sequences [50,51]. In some cases, chimeric transcription units are formed and regulated by flanking transposon sequence [52–54]. We found that *At2g10410* hypomethylation in the expressed Col allele is limited to the region of transcription (Figure 3). In addition, preliminary results suggest that flanking transposons are not expressed in genetic backgrounds expressing *At2g10410*. Because *At2g10410* is present in a transposon-rich heterochromatic pericentromere, expression at this locus may not be adequate to reverse the silenced chromatin state of the genomic region. It is possible that expression or cytosine methylation at other *Sadhu* family members present in more euchromatic, gene-rich environments may influence the expression of neighboring genes.

Non-coding transcripts such as the *Sadhu* sequences have been discovered recently in a variety of organisms, from *Drosophila* to humans to *Arabidopsis* [55,56]. In some cases, these sequences are conserved in related species and may therefore be functional. There are no related sequences to the *Sadhu* sequences in any of the currently available plant genome sequence releases, suggesting that this family is rapidly evolving. In fact, there are variable numbers of given elements even among A. thaliana accessions (Table 1 and Table 4). We are currently searching for evidence of *Sadhu*-like sequences in the genomes of other Brassicaceae species. Related sequences encoding autonomous retroelements, for instance, may provide clues to the origin of this non-autonomous retroposon family.

Finally, while *Sadhu* elements are by themselves non-protein coding, at least one element, *At1g30835,* has been incorporated into a transcribed gene (expressed in antisense orientation to other family members) capable of encoding a 132–amino acid protein. Indeed, retroelement movement is thought to increase protein coding diversity, either through incorporation into new genes [57] or by shuffling of existing exonic sequences around the genome [58–60]. We believe that the *Sadhu* retroposons, in addition to being a reservoir of transcriptional variation, may serve as genetically important wells of novel genes and gene functions.

## Materials and Methods

### Plant materials.

Col, Ler, and Cvi accessions were obtained by Lehle Seeds (Tucson, Arizona, United States); all other accessions were obtained from Arabidopsis Biological Resource Center (ABRC). Stock numbers are listed in Table 1. *ddm1–1, ddm1–2,* and *met1–1* mutants were originally isolated in the Col strain and have been introgressed greater than five generations into Col or Ler genetic backgrounds [10,28,38]. Col/Ler recombinant inbred lines that had been self-fertilized eight generations [32] were obtained from ABRC. The fifteen lines used in this study that were homozygous Ler at markers linked to *At2g10410* are CS1900, CS1911, CS1913, CS1929, CS1953, CS1954, CS1957, CS1959, CS1960, CS1968, CS1969, CS1970, CS1971, CS1974, and CS1988. The fifteen lines homozygous Col at markers linked to *At2g10410* are CS1901, CS1903, CS1904, CS1909, CS1915, CS1916, CS1932, CS1945, CS1946, CS1948, CS1951, CS1963, CS1975, CS1978, and CS1984. The Col parent line is CS933, while the Ler parent line is CS20.

Plants were grown on soil or on 1× MS media with 1% sucrose. For 5-aza-dC treatment, seedlings were germinated on 1x MS media supplemented with 1% sucrose and 10 μg/ml 5-aza-dC. DNA hypomethylation by 5-aza-dC treatment was monitored by examination of cytosine methylation at the normally methylated 180 bp repeats and 25S rRNA repeats by DNA gel blot analysis as described previously [27]. RNA or DNA was extracted from 4–6-wk-old rosette leaves or from whole 3-wk-old seedlings.

For microarray analysis, seeds were surface-sterilized and plated on 1× MS salts, 0.8% phytagar (Gibco BRL), 1× Gamborg's B5 vitamin mix, and 3% sucrose. Petri plates were incubated vertically for 14 d within a Conviron growth chamber maintained at 21 ^°^C under a 16 h light–8 h dark cycle with a light intensity of 150–175 μmol · m^−2^ · sec^−1^. Whole plant tissue samples were collected and frozen in liquid nitrogen until the RNA was extracted.

### RNA isolation and microarray hybridization.

A detailed description of RNA isolation, labeling, and hybridization protocols can be found at http://www.ag.arizona.edu/microarray. Each biological replicate consisted of approximately 50 pooled seedlings. Total RNA was isolated using TRIZOL (Invitrogen, Carlsbad, California, United States), and poly(A^+^) mRNA was purified from 75 μg of total RNA using DynaBeads Oligo (dT)25 (Dynal AS, Oslo, Norway) according to manufacturer's instructions. Purified poly (A^+^) mRNA was labeled using either Cy3- or Cy5-dUTP (Amersham Pharmacia Biotech, Piscataway, New Jersey, United States) with Clontech Powerscript reverse transcriptase (Clontech, Mountain View, California, United States). The labeled products were purified using a Millipore Microcon YM30 column (Millipore, Billerica, Massachusetts, United States), washed five times with 100 μl TE, and the final product was eluted in 40 μl TE.

For the comparison of A. thaliana accessions, we employed long oligonucleotide microarrays provided by the Galbraith laboratory (University of Arizona, Tucson, Arizona, United States; http://www.ag.arizona.edu/microarray), which are produced from a set of 26,088 single stranded 5′ amino-modified oligonucleotides, each 70 bases in length (Qiagen-Operon, Valencia, California, United States, http://oligos.qiagen.com/arrays/omad.php). These oligos have been designed to contain less than 70% homology with any other gene, minimal secondary structure, and to have a single melting temperature of at least 70 ^°^C to permit stringent microarray hybridization and washing. Each microarray element was printed once so that all genes could be accommodated on a single slide.

Total RNA from four biological replicates from each of three accessions (Ler, Cvi, and Col) were split in half before being converted to targets, resulting in eight targets from each accession (3 × 8 = 24 total targets). Targets were hybridized pair-wise in a three-sided loop design (Ler-Col, Ler-Cvi, and Cvi-Col), giving a total of 12 slides hybridized.

### Microarray data acquisition and analysis.

Hybridized slides were scanned using a GSI Lumonics ScanArray 3000 (Packard BioChip Technologies, Billerica, Massachusetts, United States). Image processing, including spot finding and quantification of signal intensity, was done using ImaGene 5.0 (BioDiscovery, El Segundo, California, United States). The median fluorescence intensity values for each spot were log base 2 transformed and normalized using the quantile method [61]. No background correction was required. The normalization effectively reduced nonlinear and linear biases due to differential incorporation of dyes, differences between slides, and effects of scanning. Linearity of the data was checked for each pairwise comparison of accessions across all oligos using least-squares means from the final analysis in an RI plot [62]. The remaining gene-specific effects and inference of differential expression among accessions was handled in a mixed-model analysis of variance (ANOVA) [63]. On an element-by-element basis, accession and dye were modeled as fixed effects, and slide modeled as a random effect (SAS Proc Mixed; SAS 8.2, SAS Institute). We tested both the raw data and residuals from the fit model for deviations from normality and homoscedasticity, on an element-by-element basis. Because there was no evidence for non-normality or unequal variance for almost all (e.g., ~2% non-normal; unadjusted α = 0.01) of the oligos, significance was determined from F-ratios. Since the purpose of this study is to discuss the general relationships among accessions, providing the basis for future work, we employed a cutoff value of 0.001 from the raw *p*-values. This corresponds to a false discovery rate (FDR) [64] of 0.011 in this data set. After the ANOVA, post-hoc pairwise Tukey tests compared adjusted means (SAS Proc Mixed) for pairwise comparisons between accessions (α = 0.001; FDR = 0.023).

### Nucleic acid manipulation.

DNA was isolated from rosette leaves or whole 3-wk-old seedlings as previously described [65]. RNA was isolated using TRIZOL reagent (Invitrogen) following the manufacturer's instructions, followed by DNAseI treatment (Invitrogen). First strand cDNA was primed with oligo-dT(15) primer using Superscript II reverse transcriptase (Invitrogen) following the manufacturer's protocol. PCR was done using standard conditions with *Taq* DNA polymerase (Qiagen) or KT1 polymerase (Clontech). Primers within the transcribed region of cyclophilin *(At4g38740)* (400 bp amplicon from genomic DNA) [66] were used as PCR amplification controls. All control amplifications utilized the same primer concentration, template amount, and number of cycles as test amplifications. PCR products destined for DNA sequencing were pretreated with exonuclease I/ Antarctic phosphatase (New England Biolabs, Ipswich, Massachusetts, United States) for 30 min at 37 °C.

For RNA gel blot analysis, RNA was size-fractionated by electrophoresis through 1% agarose formaldehyde gels and blotted to GeneScreen (NEN DuPont) nylon membranes using capillary action and 10× SSC buffer. All hybridizations were done following the protocol of Church and Gilbert [67], and membranes were washed at 60 °C in 0.2× SSC, 0.1% SDS. Hybridization probes were radiolabeled using the random priming protocol [68], and unincorporated radionucleotides were removed by size-filtration columns. Gel blots were hybridized with an amplicon from either *At2g10410* (X1 + R1, Table S2) or cyclophilin (*At4g38740* F + R). *At2g10410* RT-PCR/CAPS analysis involved amplification from cDNA template with primers X1 and R1, followed by a *Bst*B1 (New England Biolabs) digest at 65 °C following the supplier's recommended conditions. The Col allele is cleaved with *Bst*B1, generating a 480 bp fragment plus an undetected 70 bp fragment, while the Ler allele is uncleaved (550 bp) (Figure 4). The additional higher molecular weight band detected in individuals expressing both Ler and Col alleles results from unresolved heteroduplex formation during PCR [69,70]. McrBC (New England Biolabs) digests were carried out at 37 °C overnight using the supplier's recommended conditions.

Genomic DNA from Col and Ler was modified by sodium bisulfite using the CpGenome DNA Modification Kit (Chemicon, Temecula, California, United States) according to the manufacturer's protocols. PCR products were TA-cloned into pGEM-T Easy (Promega, Madison, Wisconsin, United States). Top strand products were amplified with primers Bt1 and Bt2, while bottom strand products were amplified with primers Bb1 and Bb2 (Table S2). For Col, 16 clones were sequenced from the top strand of *At2g10410* from two independent amplifications, while 11 clones were sequenced from the bottom strand. For Ler, 17 clones were sequenced from the top strand of *At2g10410* from two independent amplifications, and 11 clones from the bottom strand. For Ler *ddm1–2,* 12 clones were sequenced from the top strand of *At2g10410* from two independent amplifications, and 12 clones from the bottom strand. As a control for efficient conversion, we sequenced four clones from each converted template in the promoter region of gene *At1g01010,* which we had previously determined to be unmethylated (H. Kuo and E. J. Richards, unpublished data); we found that nearly all cytosines (>98.5%) in these clones were converted. DNA sequencing was performed using Big-Dye Terminator Cycle Sequencing (Perkin-Elmer, Wellesley, Massachusetts, United States) protocols/reagents.

### Bioinformatics.

*Sadhu* family members and partial elements were identified based on sequence similarity to *At2g01410* using iterative searches (BLOSUM62 matrix, gapped alignment, repeat filter off) on the Arabidopsis WU-BLAST server (http://www.arabidopsis.org/wublast/index2.jsp). The NCBI BLAST server (http://www.ncbi.nlm.nih.gov/BLAST) was used to confirm lack of signification sequence similarity of *Sadhu* elements to sequences outside of A. thaliana available in the public databases. Characterization of features in the vicinity of the *Sadhu* elements was aided by the repeat masker feature on the Censor server (http://www.girinst.org/censor) [71] and the genome browser at the *Arabidopsis thaliana* Small RNA Project (ASRP) website (http://asrp.cgrb.oregonstate.edu/cgi-bin/gbrowse/thaliana-v5). RT-PCR, microarray and RNA gel blot characterization of expression of *At2g10410* and other *Sadhu* elements was supplemented by reference to the Arabidopsis Tiling Array Transcriptome Express Tool (http://signal.salk.edu/cgi-bin/atta) [31], the Arabidopsis MPSS database (http://mpss.udel.edu/at/?) [30], and BLASTn to the EST database on the NCBI server (http://www.ncbi.nlm.nih.gov/BLAST). Weblogo (http://weblogo.berkeley.edu/logo.cgi) [72] was used to generate the logo image in Figure 5C. The chromosome map tool at the TAIR website (http://arabidopsis.org/jsp/ChromosomeMap/tool.jsp) aided in generating Figure S3. The maximum parsimony phylogenetic tree in Figure 5A was generated using PAUP* 4.0 (http://paup.csit.fsu.edu/about.html) based on a ClustalX alignment (ftp://ftp-igbmc.u-strasbg.fr/pub/ClustalX). Updated annotations of the sequences of *Sadhu* family members have been submitted to The Arabidopsis Information Resource (TAIR) (http://arabidopsis.org). Sequence information for all elements listed in Table 3 and Table S1 are available upon request.

## Supporting Information

Figure S1 Alignment of Col and Ler Genomic Sequence in 1.7-kb Region Encompassing *At2g10410*
Positions of the 5′ and 3′ ends of the transcript in Col is indicated. Polymorphisms are highlighted in red.
(1.5 MB EPS)

Figure S2 Diagram of Bisulfite-Mediated Genomic Sequencing at (A) Ler and (B) Col Alleles of *At2g10410* (Positions −105 to +276) The start of transcription is indicated. The number of circles above a given C indicates the number of clones sequenced; the filled circles denote the proportion of clones which were methylated. CpG sites are indicated in red, CpHpG sites in pink, and CpHpH in blue (where H = A, C, or T).
(6.8 MB EPS)

Figure S3 Map Position of 14 Full-Length (Red) and 25 Partial (Blue) *Sadhu* Elements on the Five Chromosomes of the A. thaliana Col Genome Partial elements are marked by their closest gene I.D. number. CEN = position of physical centromere.
(1.3 MB EPS)

Table S1 List of Partial *Sadhu* Elements in the Col Genome (54 KB DOC)

Table S2 Primers Used in This Study (47 KB DOC)

### Accession Numbers

The GenBank (http://www.ncbi.nlm.nih.gov/Genbank) accession numbers for the *At2g10410* genomic region sequences in Ler and Ra-0 strain backgrounds are DQ385059 and DQ385062, respectively.

## Abbreviations

MPSS: massively parallel signature sequencing
RI: recombinant inbred
tpm: transcripts per million
