# Supplementary material for: Meiotically Stable Natural Epialleles of Sadhu, a Novel Arabidopsis Retroposon
Source: PLoS Genet. 2006 Mar 17;2(3):e36. doi: 10.1371/journal.pgen.0020036 (PMC1401498; doi:10.1371/journal.pgen.0020036)
Supplement: Supplementary file 1 [file pgen.0020036.st001.doc]

| **Table S1.** List of partial *Sadhu* elements in the Col genome | | | | |
| --- | --- | --- | --- | --- |
| **Nearest Gene ID** | **Length (bp)** | **Location of fragment relative to full-length elements**  **(5’, 3’ or internal)** | **Closest full-length Sadhu element (% identity)** | **Repetitive elements within**  **2 kb** |
|  |  |  |  |  |
| *At1g18190* | 745 | 3’, with 12 bp target site duplication and poly(A) tract | *At4g01525* (99) | √ |
| *At1g32450* | 318 | 3’ | *At1g44935* (68) | √ |
| *At1g35250* | 226 | internal | *At3g02515* (70) | √ |
| *At1g36740* | 518 | internal | *At3g13438* (60) | √ |
| *At1g52615* | 349 | 3’, with poly(A) tract | *At1g50735* (66) |  |
| *At1g66790* | 248 | internal | *At5g28626* (68) |  |
| *At1g69360* | 284 | 3’ | *At1g44935* (63) |  |
| *At2g05025* | 311 | 5’ | *At5g28913* (83) | √ |
| *At2g10930* | 692 | 5’ | *At3g02515* (73) | √ |
| *At2g18530* | 243 | 3’, with poly(A) tract | *At1g35112* (76) |  |
| *At2g21900* | 564 | internal | *At3g44042* (77) |  |
| *At2g24740* | 746 | 3’, with poly(A) tract | *At1g50735* (63) | √ |
| *At3g58870* | 700 | 5’ | *At3g13438* (68) |  |
| *At3g61620* | 362 | 3’, with poly(A) tract | *At3g02515* (69) |  |
| *At4g00955* | 616 | 3’, with poly(A) tract | *At4g01525* (90) | √ |
| *At4g04920* | 583 | 3’, with poly(A) tract | *At1g03420* (62) | √ |
| *At5g03200* | 174 | internal | *At3g44042* (88) |  |
| *At5g27925* | 757 | 3’, with poly(A) tract | *At4g01525* (83) | √ |
| *At5g38910* | 201 | internal | *At1g50735* (74) | √ |
| *At5g42090* | 379 | 3’, with poly(A) tract | *At3g02515* (99) |  |
| *At5g42235* | 416 | 3’ | *At3g02515* (76) |  |
| *At5g42940* | 178 | internal | *At1g50735* (66) |  |
| *At5g44570* | 540 | 3’, with 10 bp target site duplication and poly(A) tract | *At3g02515* (77) | √ |
| *At5g46390* | 393 | internal | *At3g31442* (64) |  |
| *At5g52140* | 186 | 3’, with poly(A) tract | *At3g31442* (73) |  |
|  |  |  |  |  |
