# Supplementary material for: Meiotically Stable Natural Epialleles of Sadhu, a Novel Arabidopsis Retroposon
Source: PLoS Genet. 2006 Mar 17;2(3):e36. doi: 10.1371/journal.pgen.0020036 (PMC1401498; doi:10.1371/journal.pgen.0020036)
Supplement: Supplementary file 2 [file pgen.0020036.st002.doc]

| **Table S2.** Primers used in this study | | |
| --- | --- | --- |
| **Gene name** | **Primer name** | **Sequence (5’ 🡪 3’)** |
|  |  |  |
| *At2g10410* | Bb1 | ATG GYT GYT GTG AGG AGA G |
| *At2g10410* | Bb2 | TAT TTT AAC AAT TTA TCT CTC ATA CTA RTT ATR C |
| *At2g10410* | Bt1 | CCT AAA RAA TCC ATT CCA ATC C |
| *At2g10410* | Bt2 | AAG YAA ATT AAG AGG TGG AAY TTT TYT ATA G |
| *At2g10410* | R1 | GGA AAA GGG AGA ACC GCC AC |
| *At2g10410* | X1 | CCG CCT CTG TAA GGA GCT TC |
| *At2g10410* | X2 | ATG CTT GGA AAA CTA ATA CTA TCA AAC |
| *At2g10410* | X3 | CAA GAT CTT TGT GAA GAG AAT GTG G |
| *At2g10410* | X4 | TTG AAT TCC ACT GAG TAG GAA CAT C |
| *At2g10410* | Y1 | GCA GTT CTT TAG CTT AAT GGC CCA C |
| *At2g10410* | Y2 | CGC CCA CTC CCG TGT CAG |
| *At2g10410* | Y3 | CCA CGG TAT CTC CTA CTA GTC |
| *At2g10410* | Y4 | ACC CAG ATA AAG GAT ATA ACC TAT TTT TGA |
| *At1g30835* | F1 | AAT TTA TTG CGG ACA AGA CTC GAA G |
| *At1g30835* | R1 | GAT GGG CTG TAG AGT TAA GTG GTG |
| *At1g35112* | F1 | GAT CTT CAT AGG GGC TAA TGG TG |
| *At1g35112* | R1 | TCT GCA TCT TGA TAG TGA CCG G |
| *At3g42658* | F1 | CGA CAG CTG CGT ATG GTT AAT C |
| *At3g42658* | R1 | TCC AAT AGT ACC AGC GAT TAC AAG |
| *At3g44042* | F1 | GGA GAA TTC TCG TGG CTC AAC |
| *At3g44042* | R1 | GAC GCG AGA TCG GAT TTG GAG |
| *At5g28626* | F1 | CTT GAT GGG TTG GAG GGT GAA AC |
| *At5g28626* | R1 | AAT CCA TCA ACT ACC GAG ATC CG |
| Cyclophilin (*At4g38740*) ^a^ | F | CGA TAA GAC TCC CAG GAC TGC CGA GAA |
| Cyclophilin (*At4g38740*) ^a^ | R | TCG GCT TTC CAG ATG ATG ATC CAA CCT |
|  |  |  |

^a^	[66]

Primers corresponding to *Sadhu* elements not listed above and for the bisulfite sequencing control region are available upon request.
